# Supplementary material for: Mutations in PCYT2 disrupt etherlipid biosynthesis and cause a complex hereditary spastic paraplegia
Source: Brain. 2019 Oct 22;142(11):3382–97. doi: 10.1093/brain/awz291 (PMC6821184; doi:10.1093/brain/awz291)
Supplement: awz291_Supplementary_Data [file awz291_supplementary_data.zip › awz291-Suppl_data/Supplementary_Data4.zip!/Supplementary information 4 - plasma lipidomics.zip!/Plasma lipidomics/Control vs Patient3b/Statistical plots and data/TG species/Random forest.pdf]

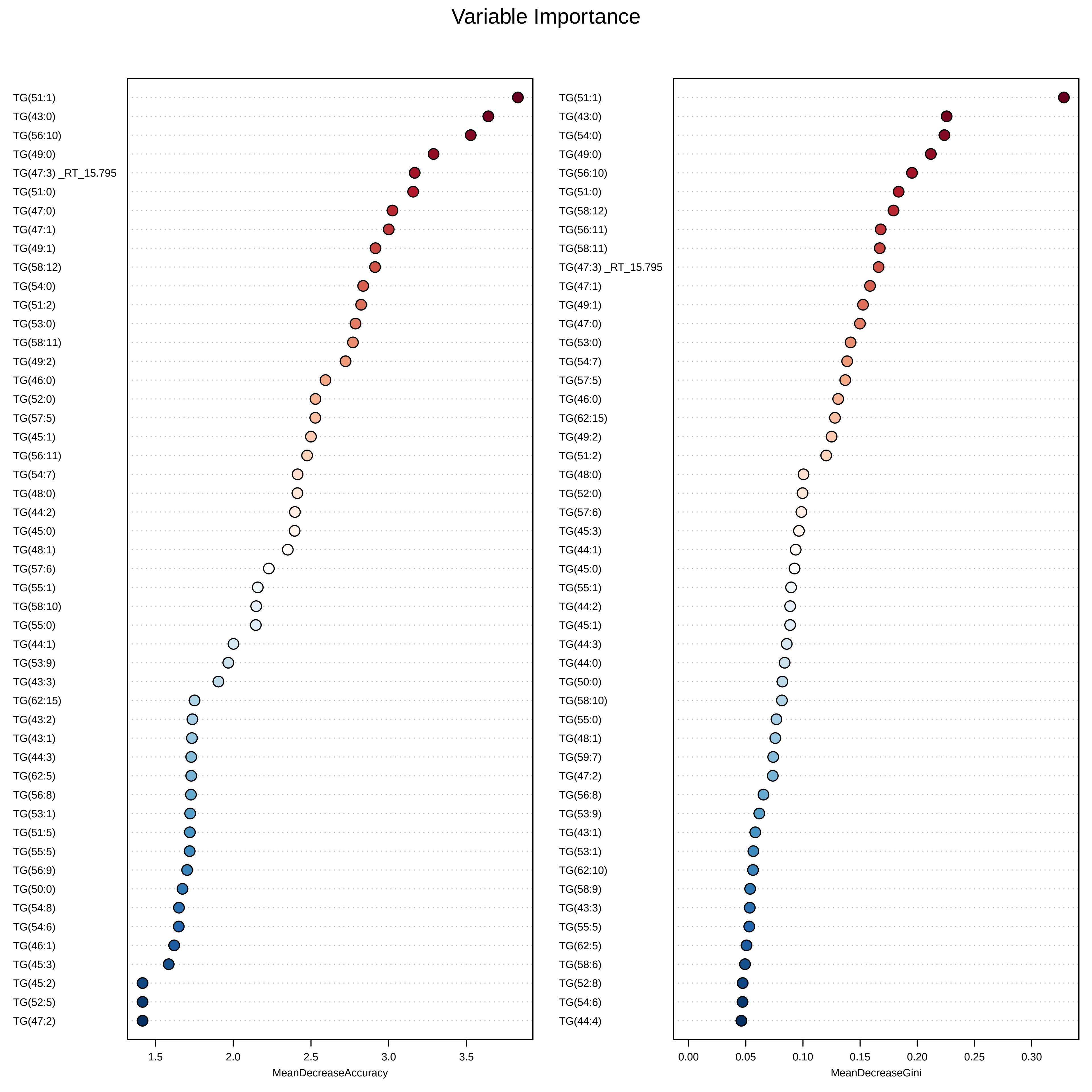

Variable Importance
TG(51:1)
TG(43:0)
TG(56:10)
TG(49:0)
TG(47:3) _RT_15.795
TG(51:0)
TG(47:0)
TG(47:1)
TG(49:1)
TG(58:12)
TG(54:0)
TG(51:2)
TG(53:0)
TG(58:11)
TG(49:2)
TG(46:0)
TG(52:0)
TG(57:5)
TG(45:1)
TG(56:11)
TG(54:7)
TG(48:0)
TG(44:2)
TG(45:0)
TG(48:1)
TG(57:6)
TG(55:1)
TG(58:10)
TG(55:0)
TG(44:1)
TG(53:9)
TG(43:3)
TG(62:15)
TG(43:2)
TG(43:1)
TG(44:3)
TG(62:5)
TG(56:8)
TG(53:1)
TG(51:5)
TG(55:5)
TG(56:9)
TG(50:0)
TG(54:8)
TG(54:6)
TG(46:1)
TG(45:3)
TG(45:2)
TG(52:5)
TG(47:2)
1.5
2.0
2.5
3.0
3.5
MeanDecreaseAccuracy
TG(51:1)
TG(43:0)
TG(54:0)
TG(49:0)
TG(56:10)
TG(51:0)
TG(58:12)
TG(56:11)
TG(58:11)
TG(47:3) _RT_15.795
TG(47:1)
TG(49:1)
TG(47:0)
TG(53:0)
TG(54:7)
TG(57:5)
TG(46:0)
TG(62:15)
TG(49:2)
TG(51:2)
TG(48:0)
TG(52:0)
TG(57:6)
TG(45:3)
TG(44:1)
TG(45:0)
TG(55:1)
TG(44:2)
TG(45:1)
TG(44:3)
TG(44:0)
TG(50:0)
TG(58:10)
TG(55:0)
TG(48:1)
TG(59:7)
TG(47:2)
TG(56:8)
TG(53:9)
TG(43:1)
TG(53:1)
TG(62:10)
TG(58:9)
TG(43:3)
TG(55:5)
TG(62:5)
TG(58:6)
TG(52:8)
TG(54:6)
TG(44:4)
0.00
0.05
0.10
0.15
0.20
0.25
0.30
MeanDecreaseGini
